# Inflammation in Renal Diseases: New and Old Players

**DOI:** 10.3389/fphar.2019.01192

**Published:** 2019-10-08

**Authors:** Vinicius Andrade-Oliveira, Orestes Foresto-Neto, Ingrid Kazue Mizuno Watanabe, Roberto Zatz, Niels Olsen Saraiva Câmara

**Affiliations:** ^1^Bernardo’s Lab, Center for Natural and Human Sciences, Federal University of ABC, Santo André, Brazil; ^2^Laboratory of Transplantation Immunobiology, Department of Immunology, Institute of Biomedical Sciences, University of São Paulo, São Paulo, Brazil; ^3^Renal Division, Department of Clinical Medicine, Faculty of Medicine, University of São Paulo, São Paulo, Brazil; ^4^Nephrology Division, Federal University of São Paulo, São Paulo, Brazil

**Keywords:** inflammation, chronic kidney disease (CKD), NF- kappa B, gut microbiota, acute kidney injury (AKI)

## Abstract

Inflammation, a process intimately linked to renal disease, can be defined as a complex network of interactions between renal parenchymal cells and resident immune cells, such as macrophages and dendritic cells, coupled with recruitment of circulating monocytes, lymphocytes, and neutrophils. Once stimulated, these cells activate specialized structures such as Toll-like receptor and Nod-like receptor (NLR). By detecting danger-associated molecules, these receptors can set in motion major innate immunity pathways such as nuclear factor ĸB (NF-ĸB) and NLRP3 inflammasome, causing metabolic reprogramming and phenotype changes of immune and parenchymal cells and triggering the secretion of a number of inflammatory mediators that can cause irreversible tissue damage and functional loss. Growing evidence suggests that this response can be deeply impacted by the crosstalk between the kidneys and other organs, such as the gut. Changes in the composition and/or metabolite production of the gut microbiota can influence inflammation, oxidative stress, and fibrosis, thus offering opportunities to positively manipulate the composition and/or functionality of gut microbiota and, consequentially, ameliorate deleterious consequences of renal diseases. In this review, we summarize the most recent evidence that renal inflammation can be ameliorated by interfering with the gut microbiota through the administration of probiotics, prebiotics, and postbiotics. In addition to these innovative approaches, we address the recent discovery of new targets for drugs long in use in clinical practice. Angiotensin II receptor antagonists, NF-ĸB inhibitors, thiazide diuretics, and antimetabolic drugs can reduce renal macrophage infiltration and slow down the progression of renal disease by mechanisms independent of those usually attributed to these compounds. Allopurinol, an inhibitor of uric acid production, has been shown to decrease renal inflammation by limiting activation of the NLRP3 inflammasome. So far, these protective effects have been shown in experimental studies only. Clinical studies will establish whether these novel strategies can be incorporated into the arsenal of treatments intended to prevent the progression of human disease.

## Introduction

Acute kidney injury (AKI) and chronic kidney disease (CKD) are highly prevalent conditions associated with significant levels of morbidity and mortality. According to the US Renal Data System, about 30 millions of Americans were afflicted with CKD in 2017, a prevalence of almost 15% (Saran et al., 2017; Saran et al., 2018). As to AKI, recent evidence indicates that it affects almost 60% of intensive care unit (ICU) patients, whereas its severity is directly related to mortality (Hoste et al., 2015).

Although the mechanisms underlying CKD and AKI are quite distinct, clinical evidence suggests that the two conditions are inextricably interconnected (Chawla et al., 2014). Inflammation, a process aimed in principle at detecting and fighting harmful pathogens, is a major pathogenic mechanism for both CKD and AKI. Both resident and circulating immune cells can interact with parenchymal renal cells to trigger an inflammatory response when subjected to stress, causing irreversible tissue damage and eventuating in organ failure (Singbartl et al., 2019). Thus, targeting inflammation constitutes a rational strategy in the management of both CKD and AKI.

## Kidney Resident Immune Cells

**Figure 1:**
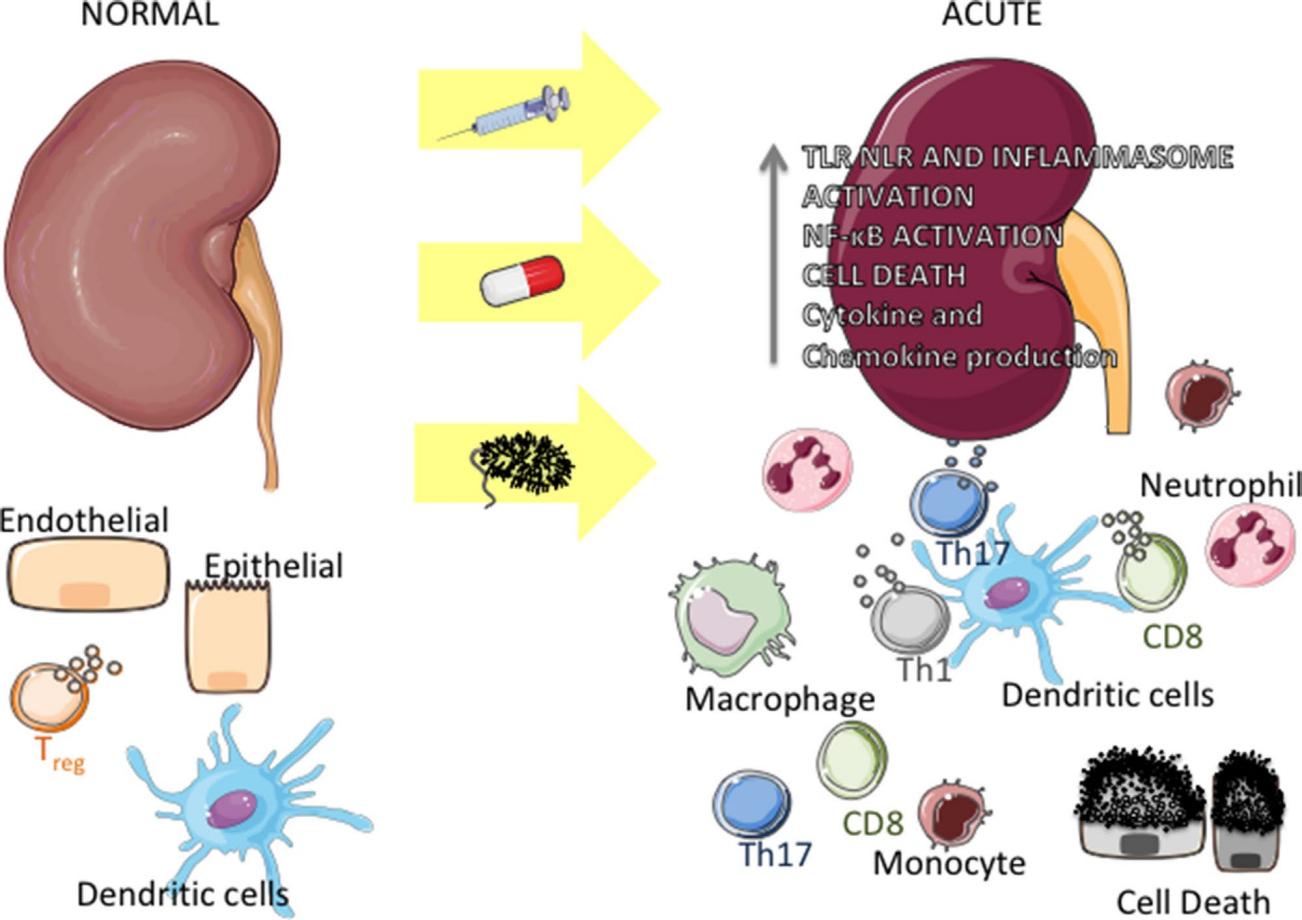
Inflammation in acute kidney diseases. Under physiologic conditions, endothelial, epithelial, and immune cells (around parenchymal structures and/or vessels) interact harmonically within the kidney (left side). Upon an insult by bacteria or bacterial products, drug toxicity, or following nonsterile stimulation, epithelial and endothelial cells undergo necrosis or apoptosis, releasing products that can activate Toll-like receptors (TLR), NOD-like receptors (NLR), and NLPR3 inflammasome in immune and kidney cells. This activation leads to the production of chemokines and proinflammatory cytokines, which recruit monocytes and neutrophils to the organ. Concomitantly, resident immune cells (mainly dendritic cells) get activated and induce the proliferation of T cells (T_H_1, T_H_17, and CD8 cytotoxic cells), which in turn produce cytokines, exacerbating the inflammation process (right).

The kidney harbors a variety of resident immune cells, which play an important role in the maintenance of tissue homeostasis. Dendritic cells (DCs), macrophages, regulatory T cells (Tregs), CD8, and NK lymphocytes, among other cell types, are in close contact with parenchymal cells (Kurts et al., 2013; Stamatiades et al., 2016) (**Figure 1**, left side). Once activated by external (for instance, microbial antigens) or by internal events, these cells produce inflammatory mediators that can initiate kidney disease and, concomitantly, trigger a regulatory response aimed at curbing inflammation, repairing tissue damage, and restoring homeostasis. For instance, microbial products and alarmins can bind to innate immune receptors on the surface of DC, leading these cells to secrete a number of chemokines and cytokines (Dong et al., 2007; Tittel et al., 2011) and to migrate to draining lymph nodes, where they can amplify the immune response by activating T lymphocytes (T_H_1 CD4^+^ and cytotoxic cells). At the same time, DCs can limit inflammation by stimulating the production of interleukin 10 (IL-10) by Treg lymphocytes (Kinsey et al., 2009; Monteiro et al., 2009). Another clear example of this dichotomous activity is given by macrophages, which, by sensing alarmins through innate immune receptors, can initiate/enhance inflammation *via* the M1 subset and, simultaneously, exert an anti-inflammatory effect *via* the M2 subset (Nelson et al., 2012;Braga et al., 2017a) (**Figure 1**, right side).

## Inflammation in AKI

### Role of Toll-Like Receptor– and Nod-Like Receptor–Dependent Pathways

Ischemia–reperfusion injury (IRI) is a major cause of AKI in ICU patients (Hoste et al., 2015). Transcriptome analysis in whole tissue as well as cell-specific analysis using Cre/Lox techniques revealed that IRI is associated with a myriad of events ranging from organelle stress to activation of complex intracellular pathways (Correa-Costa et al., 2012; Liu et al., 2014). One of the earliest events in the development of IRI is sterile (nonmicrobial) activation of innate immune receptors such as TLRs (Toll-like receptors) and NLRs (Nod-like receptors). These structures, expressed in renal parenchymal cells as well as in resident immune cells, can recognize pathogen-associated molecular patterns (PAMPs) and damage-associated molecular patterns (DAMPs). Activation of these receptors triggers a number of intracellular pathways such as c-Jun N-terminal kinases (JNK), mitogen-activated protein kinase (MAPK), and nuclear factor kB (NF-ĸB), culminating with the secretion of proinflammatory cytokines and chemokines (Wang and Zhang, 2017). Much of the current knowledge about the role of these receptors in AKI came from studies of knockout (KO) mice. Lack of TLR4 and/or TLR2, as well as of their adaptor molecule, *myeloid differentiation factor 88 (MyD88)*, prevented the development of kidney injury in mice undergoing IRI (Leemans et al., 2005; Shigeoka et al., 2007; Wu et al., 2007). Available evidence indicates that activation of these receptors and the development of downstream inflammatory events follow the binding of alarmins released after cell injury and/or death (Rosin and Okusa, 2011; Allam et al., 2012). Accordingly, upregulation and blockage of one of these alarmins, high-mobility group box 1, was associated with aggravation and lessening of renal injury, respectively (Wu et al., 2010; Chen et al., 2017). A similar mechanism seems to be operative in murine models of sterile (cisplatin toxicity) and nonsterile (sepsis) AKI (Castoldi et al., 2012; Andrade-Silva et al., 2018). Castoldi et al. (Castoldi et al., 2012) showed that lack of TLR4, TLR2, and *MyD88* limited renal damage in a sepsis model, mainly by decreasing the recruitment of neutrophils. In mice with cisplatin-induced AKI, we showed evidence that the presence of TLR4 is needed for the development of renal damage (Andrade-Silva et al., 2018), an action that can be mediated by p38 MAPK pathways (Zhang et al., 2008). Additional evidence of participation of these receptors is provided by deceased-donor renal grafts, in which the inevitable IRI is associated with increased expression of TLR4 (Kruger et al., 2009; Andrade-Oliveira et al., 2012). Conversely, a mutation in the tlr4 gene decreases the signaling through TLR4 receptor, which may attenuate the response of kidney grafts to alarmins, hence limiting cytokine production, inflammation, and eventual organ failure (Nogueira et al., 2010).

Inflammation initiated in the kidneys can lead to the release of cytokines and chemokines to the circulation, resulting in dysfunction of distant organs such as heart and lungs (Kramer et al., 1999; Campanholle et al., 2010; Rostron et al., 2010; Trentin-Sonoda et al., 2015). Thus, activation of renal TLR4 sets off an important signaling pathway that acts both locally and at remote organs, contributing to build up a state of systemic inflammation that considerably aggravates the clinical scenario. TLR4 blockade or inactivation constitutes a promising target for the prevention or mitigation of AKI.

Whereas TLRs can be located on the surface of cells and endosomes, NLRs are equally complex intracellular sensors that are expressed throughout the kidney and have been associated with several models of AKI. On the basis of their structure, NLRs are subdivided into three subgroups, namely, NOD1 and NOD2 or NLRP (Latz et al., 2013). Activation of NOD1/NOD2 sets in motion the NF-ĸB and MAPK cascades (Carneiro et al., 2008), whereas that of NLRP leads to cleavage of IL-1β and IL-18 (Wen et al., 2013). Shigeoka et al. (Shigeoka et al., 2010a) showed that, when subjected to renal ischemia, NOD2 KO and NOD1/NOD2 double KO mice develop less renal inflammation and tubular cell apoptosis than wild mice, being thus protected from IRI. Similar protection against tubular cell death and renal functional loss was observed in NLRP3 KO mice subjected to renal ischemia (Iyer et al., 2009; Shigeoka et al., 2010b; Kim et al., 2013). Together, these studies lend support to the notion that the NLRP3 pathway plays a key role in the pathogenesis of inflammation and cell death in IRI-associated AKI. The participation of NLRP3 may be less prominent in other modalities of AKI, since NLRP3 KO mice were not protected against kidney damage induced by cisplatin (Kim et al., 2013).

### Immune Cells in AKI

#### Monocytes and Macrophages

In mice, circulating monocytes can be subdivided into inflammatory (Cx3CR1^low^, CCR2^+^, and Ly6C^high^) and resident (Cx3CR1^high^, CCR2^neg^, and Ly6C^low^) cells (Geissmann et al., 2003). Ly6C^high^ monocytes protect the kidney from damage induced by sepsis, presumably by sensing and orchestrating a response against bacterial proliferation. Ly6C^low^ monocytes are located at the perivascular space. Their interaction with endothelial cells decreases intercellular adhesion molecule 1 expression, thus preventing neutrophil infiltration and inflammation in IRI (Karasawa et al., 2015).

Macrophage depletion in rats protects kidney function from IRI injury (Jo et al., 2006), whereas blockade of the migration inhibitory factor was shown to inhibit macrophage infiltration and to ameliorate inflammation in cisplatin-induced AKI (Li et al., 2018). These findings suggest that macrophages exert a central pathogenic role in this setting. However, others reported opposite results (Lu et al., 2008). The reason for this discrepancy is unclear but may be related to the existence of two different macrophage populations. As pointed out earlier, monocytes can differentiate into M1 or M2 macrophages, which are induced by distinct factors and acquire distinct functions (Wang et al., 2014). M1 macrophages appear early in the course of kidney IRI, suggesting that they contribute to initial injury, whereas the presence of M2 macrophages is predominantly seen at later phases, presumably as part of a repair process (Lee et al., 2011). Interestingly, transference of M1 macrophages into macrophage-depleted mice subjected to IRI rescued the adverse phenotype observed in control animals (Lee et al., 2011). Thus, macrophages, mainly M1, play an important pathogenic role at the onset of AKI. The existence of other macrophage subsets, their plasticity, and their role in AKI models are currently the object of intense investigation.

#### Neutrophils

Renal infiltration by neutrophils can be detected as early as 2 h after IRI, remaining elevated up to 72 h after the insult (Awad et al., 2009). Upon reaching the renal parenchyma, neutrophils are exposed to DAMPs and PAMPs, leading to activation of TLRs and NLRs (Castoldi et al., 2012; Deng et al., 2017) and to the secretion of a number of inflammatory factors such as proteases, reactive oxygen species (ROS), and lytic enzymes (Leliefeld et al., 2016). These compounds interact with the vascular endothelium, leading to vessel tumefaction and, consequently, impairing blood flow (Jerke et al., 2015). Accordingly, neutrophil depletion exerts a protective effect in several AKI models (Thornton et al., 1989; Castoldi et al., 2012). Recently, Raup-Konsavage et al. (Raup-Konsavage et al., 2018) showed that the formation of neutrophil extracellular traps (NETs), which are instrumental for neutrophils to kill microorganisms (Brinkmann et al., 2004), could be one of the mechanisms by which these cells cause tissue damage during AKI. They showed that mice KO for PAD4, an enzyme involved in NET formation, exhibited partial preservation of kidney function after IRI (Raup-Konsavage et al., 2018). However, it must be stressed that these harmful effects constitute collateral damage and that, since the primary function of neutrophils is to fight infection, their absence can be detrimental. In the model of sepsis by cecal ligation and puncture, Hoesel et al. (Hoesel et al., 2005) showed that neutrophil depletion at the onset of sepsis can impair survival due to loss of antibacterial power and that a protective effect is achieved only when neutrophil depletion is performed at a later phase.

Since neutrophils appear so early in AKI, neutrophil-related proteins such as neutrophil gelatinase-associated lipocalin can be used as biomarkers of kidney damage and potential predictors of renal dysfunction and survival, as for instance in critically ill patients and in recipients of renal grafts (Parikh et al., 2006; Valette et al., 2013).

#### Dendritic Cells

DCs are mononuclear phagocytes that, as resident cells in several organs, perform a variety of functions, acting as a bridge between innate and adaptive immunity. In the kidneys, DCs heterogeneous population, exerting several functions, such as antigen-presenting, T-cell stimulation, and production of anti-inflammatory IL-10, (Kawakami et al., 2013). Kidney DCs are located between tubules and peritubular capillaries, allowing them to interact with effector cells as well as with endothelial and epithelial cells (Lemley and Kriz, 1991). During AKI, kidney DCs are activated by binding of TLRs and NLRs to cell debris and other DAMPs, thus increasing the expression of costimulatory molecules and proinflammatory cytokines such as tumor necrosis factor α (TNF-α) and antigen presentation to T cells in draining lymph nodes (Dong et al., 2007; Snelgrove et al., 2017), thus contributing to T-cell–mediated inflammation. By contrast, renal infiltration by neutrophils and monocytes, as well as tubular injury, was worsened in DC-depleted mice undergoing cisplatin-induced AKI compared to control animals (Tadagavadi and Reeves, 2010), suggesting that, in this setting, the presence of DCs may exert renoprotective rather than deleterious effects. The reason for this unexpected effect is unclear and may be related to a modulatory effect of DCs on the inflammatory response (Tadagavadi and Reeves, 2010). Alternatively, since DCs constitute a heterogeneous population, it is conceivable that, depending on the context, one or more subtypes exert a crucial anti-inflammatory function. Another possibility would be that monocytes and neutrophils simply occupied the niche left by the missing DCs and necrotic tubular cells. These hypotheses are currently speculative and need further investigation to be tested.

#### T Cells

Several lines of evidence indicate that the adaptive immune response is also involved in the pathogenesis of AKI. Animals lacking CD4^+^ and CD8^+^ T cells are protected from IRI-induced AKI (Rabb et al., 2000; Deng et al., 2017), an effect associated with reduced neutrophil infiltration. CD4^+^ T cells can differentiate into different subsets—effector T-helper (T_H_) T_H_1/T_H_2/T_H_17 or Tregs, according to the microenvironment, to the expression of costimulatory molecules, to the presence of cytokines, and, as recently demonstrated, to variations in cell metabolism (Parish and Kaech, 2009; Zhu et al., 2010; MacIver et al., 2013). Evaluation of specific subtypes of CD4^+^ T cells in IRI-induced AKI indicates that T_H_1 cells tend to worsen kidney damage, since mice KO for IL-12, a cytokine known to induce T_H_1 cells, are protected from IRI, whereas in mice KO for IL-4, cytokine known to induce T_H_2 cells, kidney damage is exacerbated (Marques et al., 2006; de Paiva et al., 2009). Moreover, T-cell–specific deletion of STAT-3, a transcription factor associated with T_H_17 differentiation, was renoprotective in animals subjected to IRI (Lee et al., 2018). IL-17 production by T_H_17 cells is known to increase the recruitment of neutrophils. Accordingly, IL-17 KO mice undergoing sepsis exhibited limited renal recruitment of neutrophils, associated with reduced apoptosis and less severe AKI. Consistent with these findings, higher IL-17 levels have been associated with worse outcomes in septic patients (Maravitsa et al., 2016). Thus, IL-17 and T_H_17 cells are likely to exert a prominent role in kidney injury during sepsis. Tregs have also received considerable attention in the context of AKI. Kinsey et al. (Kinsey et al., 2009) showed that depletion of Tregs worsened kidney damage during IRI-induced AKI by enhancing renal infiltration of macrophages and neutrophils and by augmenting the local levels of proinflammatory cytokines. Likewise, exacerbated kidney injury and impaired renal function after Tregs depletion were reported by Monteiro et al. (Monteiro et al., 2009). The renoprotective role of Treg was also shown in cisplatin-induced AKI (Lee et al., 2010). Recently, an interesting population of T cells double negative for CD4 and CD8 was described, which may represent yet another physiologic anti-inflammatory mechanism. These cells can be detected early in the course of AKI, playing a renoprotective role in an IL-10–dependent manner (Martina et al., 2016).

## Inflammation in CKD

The mechanisms underlying the progression of CKD involve a complex interaction between hemodynamic, immunologic, metabolic, and inflammatory events (Zoccali et al., 2017). The development of inflammation, previously associated with immune dysfunction only, is currently considered a fundamental component of the pathogenesis of even non–immune-mediated CKD (Floege et al., 1992). Activation of DCs was shown to enhance the activity of CD8+ T cells, thus promoting glomerular injury in two different experimental models of progressive renal disease (Heymann et al., 2009; Cao et al., 2016). Accordingly, inhibition of DC activation or of their interaction with CD8+ T cells preserved kidney function and decreased inflammation and fibrosis in adriamycin glomerulopathy (Wang et al., 2018). Other subtypes of T cells, especially TH2, may also contribute to renal damage. Liu et al. (2012) showed massive infiltration by CD4+ T lymphocytes in advanced human immunoglobulin A nephropathy and that depletion of CD4+ T lymphocytes prevented renal fibrosis in UUO (unilateral ureteral obstruction) mice. They also showed that the TH2/TH1 ratio increased progressively after UUO and that TH2-reconstituted mice were more prone to develop renal fibrosis than TH1-reconstituted animals. Likewise, Braga et al. (2012) observed an increase in TH2-related cytokines 7 days after UUO in mice, as well as an accumulation of M2 macrophages in an MyD88 pathway–dependent manner.

**Figure 2:**
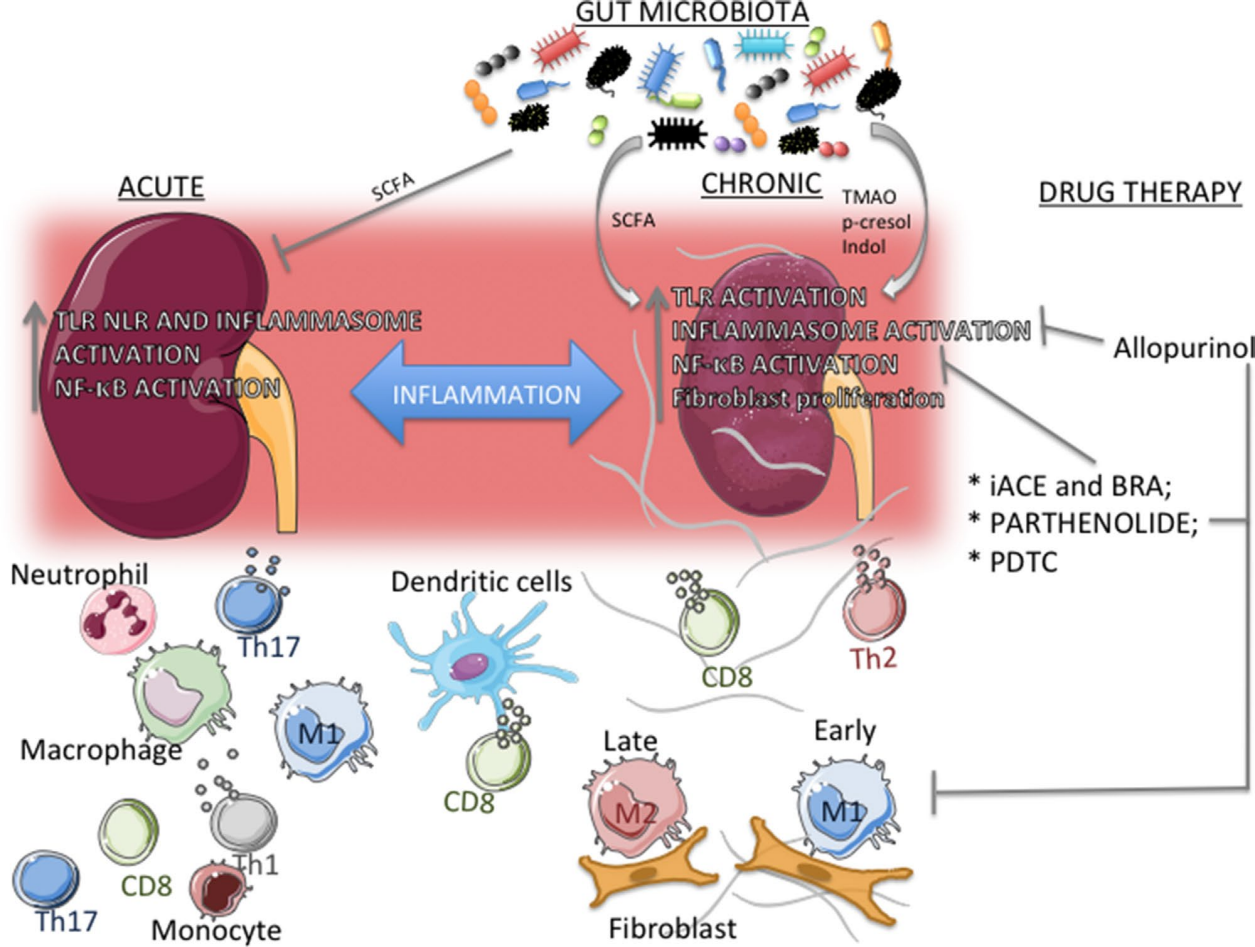
Actors in the transition from acute kidney injury (AKI) to chronic kidney disease (CKD). Inflammation is a common link between AKI and CKD, and increased expression and/or activation of TLR, NLRP3 inflammasome, and NF-ĸB are present in both scenarios. The composition of the immune cell population depends on the context. In AKI, a large number of T_H_1/T_H_17 lymphocytes and neutrophils concentrate around parenchymal structures and/or vessels, whereas in CKD T_H_2 lymphocytes and M2 macrophages predominate. M1 macrophages, dendritic cells, and CD8^+^ T cells are seen in both processes. In CKD, epithelial– and/or endothelial–mesenchymal transition, with fibroblast proliferation, may contribute substantially to the development of inflammation and fibrosis. Gut microbiota composition, along with its subproducts, can have an important role in both AKI and CKD. Short-chain fatty acid (SCFA) can inhibit inflammation in different models of AKI, whereas in CKD there is a reciprocal relationship, with gut microbiota modulating the development of CKD, and, conversely, uremic toxins (e.g., p-cresol and indol) promoting changes in the composition of gut microbiota. Repurposed drugs and compounds that limit lymphocyte proliferation, M1 infiltration, and specific inflammatory pathways can exert renoprotective and anti-inflammatory effects. Angiotensin-converting enzyme inhibitors and angiotensin II receptor blockers inhibit the NF-ĸB system, whereas the experimental compounds pyrrolidine dithiocarbamate and parthenolide provide more specific inhibition. Allopurinol reduces activation of the NLRP3 inflammasome.

The role of macrophages and that of its subtypes M1 and M2 have been extensively investigated in different models of CKD. Given their properties, the notion has been established that, in various organs and experimental models of disease, M1 macrophages act in early phases of inflammation and, as this process evolves, M2 macrophages eventually predominate to promote repairing and fibrosis (Wynn and Barron, 2010). In UUO mice, Braga et al. (2016) observed that infiltration by M1 macrophages occurs early in the course of the inflammatory process, whereas M2 macrophages accumulate only at more advanced phases, suggesting that these cells possess a resolutive, rather than profibrotic, phenotype. While this relationship between M2 macrophages and fibrosis development seems to be clear, a study using CRE-lox techniques to deplete IL-4Rα in lysozyme^+^ cells (macrophages and neutrophils), therefore preventing macrophages from interacting with IL-4 and IL-13—two type 2 cytokines responsible for macrophage M2 polarization —showed that, in such context, mice developed an extremely severe, and often fatal, T_H_1-associated inflammatory response to schistosomal infestation, whereas the reaction against a form of murine strongyloidiasis was adequate (Herbert et al., 2004). These findings suggest that, at least in some forms of inflammation, the M2 phenotype is essential not only for tissue repair but also to prevent excessive inflammation and organ damage. Together, these findings indicate that the recruitment and differentiation of macrophages are crucial to all phases of renal injury, from triggering tissue injury to tissue repair. Any disturbance of this delicate equilibrium and/or the persistence of tissue insults will tip the balance in favor of macrophages directly involved in the development of renal inflammation (Correa-Costa et al., 2014; Amano et al., 2018; Braga et al., 2018)(**Figure 2**).

The mechanisms leading from the initial insult to the development of inflammation and consequent renal fibrosis are incompletely understood and may involve, among others, mechanical stress to the glomerular walls due to intracapillary hypertension and/or tuft hypertrophy, direct chemical aggression to podocytes and other glomerular barrier elements, exposure of tubular cells to high protein concentrations in situations of intense proteinuria, and the participation of the renin–angiotensin–aldosterone system (RAAS) and of numerous cytokines, chemokines and growth factors. In recent years, the concept has been established that activation of innate immunity pathways, particularly that of the NF-ĸB system and of the NLPR3 inflammasome, is an important link between early events such as glomerular hypertension and advanced phenomena such as the development of renal fibrosis. This pathogenic association has been shown in several CKD models.

The expression of NLRP3, TLRs, and biglycan was shown to increase in mice subjected to UUO (Leemans et al., 2009; Pulskens et al., 2010; Braga et al., 2012; Pulskens et al., 2014). Conversely, MyD88 KO mice exhibited less fibrosis after UUO (Braga et al., 2012). TLR2 and TLR4, as well as the NLRP3 inflammasome, seem to be activated during the development of tubulointerstitial inflammation in mice receiving dietary adenine overload, which were protected from renal fibrosis when genes coding for these molecules were deleted (Correa-Costa et al., 2011). Accordingly, NLRP3 activation was associated with increased inflammation in a rat model of heavy proteinuria (Faustino et al., 2018). Recent data suggest that activation of these pathways persists throughout the development of CKD. In the 5/6 renal ablation model (Nx), the expression of innate immune-related molecules was highly expressed as early as 15 days and, for at least 60 days, after renal ablation (Fanelli et al., 2017).

The NF-ĸB cascade is another major intracellular pathway for the development of inflammation. In canonical NF-ĸB activation, the IkB kinase (IKK) degrades the inhibitory protein IkB, allowing heterodimers (e.g., p50/p65) to translocate from the cytosol to the nucleus, where they bind to the DNA and promote the transcription of a number of genes that code for proinflammatory mediators (Henkel et al., 1993). Evidence that the NF-ĸB system is involved in the development of renal inflammation and injury has been described in a variety of clinical and experimental renal diseases, such as diabetic kidney disease (Lee et al., 2004; Verzola et al., 2014), 5/6 renal ablation (Fujihara et al., 2007a;Fanelli et al., 2017), adenine overload (Correa-Costa et al., 2011; Okabe et al., 2013), and adriamycin nephropathy (Faustino et al., 2018).

Although diabetic nephropathy, hypertensive nephrosclerosis, and primary glomerulopathies are currently considered the main cause of end-stage renal disease (ESRD) (Saran et al., 2017), growing evidence indicates that AKI-associated renal inflammation may persist even after acute injury is overcome and renal function restored, giving rise to slowly progressive renal fibrosis (Liano et al., 2007; Venkatachalam et al., 2010; Chawla and Kimmel, 2012).

As a whole, these observations indicate that AKI and CKD share a number of inflammatory pathways whereby TLRs and NLRs are activated, along with immune cells such as macrophages, DCs, and lymphocytes. The exact mechanisms by which these cells and receptors are stimulated and the full consequences of their activation are currently under intense investigation, which is expected to provide novel therapeutic strategies for these diseases. In recent years, evidence has accumulated that the gut microbiota composition may be different in those two different diseases, and may trigger and/or amplify the inflammatory response in the kidney.

## The Mutualistic Relationship Between Microbes and Human Body

The human body harbors complex communities of commensal microbes, including bacteria, archaea, fungi, viruses, and protozoa. The gastrointestinal tract is one of the largest interfaces between the human body (host) and the environment. The composition and activity of gut microbiota start before birth (Jimenez et al., 2005; Jimenez et al., 2008;Aagaard et al., 2014) and are influenced by a variety of factors, including birth mode (Dominguez-Bello et al., 2010), breastfeeding (Martin et al., 2012), age (Yatsunenko et al., 2012), geography (Yatsunenko et al., 2012), sanitation (Gao et al., 2017), use of antibiotics (Perez-Cobas et al., 2013), and exercising (Clarke et al., 2014).

Throughout the past century, evidence was amassed that microbe–host interactions modulate a large number of vital functions in the healthy human body, including host metabolism (Nicholson et al., 2012) and immunity (Rooks and Garrett, 2016), as well as cardiovascular and brain functions. In this mutualistic relationship, the intestine provides a very favorable environment for microbes, with free access to nutrients and controlled temperature. The gut microbiota, on the other hand, is intrinsically involved in the morphogenesis and physiology of the immune system, protecting the host from potential pathogens and regulating metabolism and nutrition. Additionally, the microbial metabolism utilizes enzymes that are not encoded by the human genome and generates several biologic products essential to the host’s health, such as bile acids, choline, vitamins, and short-chain fatty acids (SCFAs) (Rowland et al., 2018).

SCFAs are the main end products of dietary carbohydrate metabolized by the gut microbiota and made available to the host. The three most abundant SCFAs, acetate, propionate, and butyrate, participate in several host vital functions. Besides being used as energy source by colonocytes, SCFAs play a key role in the maintenance of intestinal barrier integrity. Butyrate seems to regulate the expression of claudin-1 and the redistribution of zona occludens-1 and occludin, which are essential components of the tight junction assembly (Wang H et al., 2012). SCFAs have also extraintestinal actions, including appetite regulation (Frost et al., 2014), glucose and lipid metabolism (den Besten et al., 2015; Soty et al., 2015), and immune regulation (Rooks and Garrett, 2016), as shown by recent studies. Exposure of neutrophils and peripheral blood mononuclear cells to SCFAs attenuated the activation of NF-ĸB and inhibited the production of proinflammatory cytokines (Usami et al., 2008; Vinolo et al., 2011; Chang et al., 2014). SCFAs also modulate the activity of Tregs, conferring protection conditions of sustained activation of immune system (Smith et al., 2013; Thorburn et al., 2015). These functions rely on the ability of SCFAs to inhibit histone deacetylases and their actions through a group of G protein–coupled receptors, which are able to respond to short-, medium-, or long-chain free fatty acids (Hara et al., 2013). This group includes GPR41 (FFAR3), GPR43 (FFAR2), and GPR109a (HCAR2), which are responsible for the action of SCFAs in a dose-dependent manner (Brown et al., 2003) and have been described in several cell types, such as adipocytes (Ge et al., 2008; Wanders et al., 2012), neurons (Kimura et al., 2011), immune cells (Le Poul et al., 2003; Kostylina et al., 2008), and vascular cells (Hughes-Large et al., 2014).

### Kidney Diseases and Dysbiosis

Low-grade inflammation is a major component not only of renal diseases but is also common ground for a variety of other noncommunicable diseases, including cardiovascular disease, metabolic syndrome, neurological disorders, and allergic conditions (Camps and Garcia-Heredia, 2014). The development and activation of the immune system are highly dependent on microbial–host interactions, and an unbalanced relationship can lead to sustained immune activation or inappropriate suppression (Rook, 2013). Several environmental and host-related factors can disturb the microbial ecosystem to such an extent that its resilience and resistance abilities are overcome. Despite the inexistence of a core microbiome shared among healthy individuals (Human Microbiome Project, 2012; Yatsunenko et al., 2012; Lloyd-Price et al., 2016), a common bacterial core is composed of four main phyla: Actinobacteria, Proteobacteria, Firmicutes, and Bacteroidetes (Arumugam et al., 2011). Mounting evidence indicates that compositional and functional changes in the gut microbiota (dysbiosis) are associated with intestinal and extraintestinal diseases.

Although it remains unclear whether alterations in gut microbiota caused by extrinsic factors could be the primary agent to trigger kidney injury, in the past years several studies clearly showed that impaired kidney function contributes to gut dysbiosis, which in turn aggravates kidney injury. The importance of gut microbiota in kidney diseases was demonstrated by Jang et al. (2009), who showed that germ-free mice are more susceptible to kidney injury, whereas reintroduction of a gut microbiota conferred protection against the disease. Germ-free mice exhibited an abnormal high number of natural killer T cells and lower IL-4 levels in the kidneys, which were more susceptible to ischemia–reperfusion damage.

Growing evidence indicates that gut dysbiosis causes gut inflammation and impairs intestinal barrier integrity, resulting in activation of the NF-ĸB pathway and, ultimately, systemic inflammation (Anders et al., 2013). Disruption of intestinal barrier enables the translocation of bacterial endotoxins, such as lipopolysaccharide (LPS), leading to activation of immune cells at the lamina propria and to enhanced secretion of proinflammatory cytokines. Of note, endotoxemia is correlated with the risk of mortality in CKD patients (McIntyre et al., 2011). Microbial dysbiosis can also contribute to kidney failure through the accumulation of bacterial-produced uremic toxins, such as indoxyl sulfate (IS), p-cresol sulfate, and trimethylamine-N-oxide (TMAO). Using germ-free technology, Wikoff et al. (2009) demonstrated that the gut microbiome strongly influences the levels of several protein-bound uremic toxins. Under healthy conditions, these toxins are partially eliminated with the feces, whereas whatever amount absorbed by the intestine is excreted by the kidneys. Therefore, impairment of kidney function can lead to the retention of uremic toxins that originated in the gut. Several studies have observed high levels of these toxins in patients with kidney failure. Increased levels of p-cresol sulfate were found in patients with mild to moderate CKD and were associated with low estimated glomerular filtration rate (Meijers et al., 2010a). Likewise, circulating TMAO levels rise as kidney function declines in CKD patients (Stubbs et al., 2016) and correlate with lower long-term survival rates (Tang et al., 2015). Some studies reported adverse effects of uremic toxins on the kidney. These toxins instigate inflammation (Motojima et al., 2003) by increasing ROS production and the levels of proinflammatory cytokines (Watanabe et al., 2013) (**Figure 2**). In addition, they can promote renal fibrosis through reduction of klotho expression and cell senescence (Adijiang et al., 2011).

It is noteworthy that, in turn, CKD can affect the status of gut microbiota, thus contributing to dysbiosis. Growing evidence has shown significant changes in the gut microbiota of CKD patients in terms of individual species (Hida et al., 1996; Wang I et al., 2012; Vaziri et al., 2013). Kidney diseases lead to metabolic acidosis, volume overload, recurrent use of antibiotics, prolonged colonic transit time (Bammens et al., 2003), intestinal wall edema (Vaziri et al., 2013), and reduction of dietary fiber intake (Kalantar-Zadeh et al., 2002), which can not only alter the intestinal permeability, but may cause an imbalance in gut microbiota. Moreover, profound changes can be induced by uremia *per se*. Vaziri et al. (2013) observed significant alterations in the abundance of 175 bacterial operational taxonomic units in nephrectomized rats, indicating that the effect of uremia is independent from interindividual variations, comorbid conditions, and dietary and medical interventions. Furthermore, ESRD patients showed expansion of bacterial families possessing enzymes that produce uremic toxins in detriment of those expressing enzymes that degrade fibers and generate SCFAs (Wong et al., 2014). This adverse scenario is further aggravated by the medical recommendation to reduce the intake of fruits and vegetables to avoid hyperkalemia. Because fiber-rich foods are the major source of resistance starch used to generate SCFAs, one can speculate that the consequent reduction in the production of SCFAs may contribute to kidney damage. Indeed, low intake of fiber forces a switch in the gut microbiota metabolism leading to decreased production of SCFAs and to the generation of harmful metabolites (Duncan et al., 2007; Russell et al., 2011). A potential additional adverse effect of fiber restriction was pointed out in an elegant experimental study showing that, in a scenario of dietary fiber deficiency, gut microbiota resorts to host-secreted mucus glycoproteins as a nutrient source, resulting in a thinner colonic mucus layer, which could contribute to enhanced pathogen susceptibility, endotoxemia, and, ultimately, systemic inflammation (Desai et al., 2016).

### Gut Microbiota–Based Therapies

In an elegant study, Poesen et al. (2016) reported a high resemblance between the fecal metabolite profiles of CKD patients and those of their household contacts on the same diet, after adjustment for age, gender, body mass index, and the presence of diabetes mellitus. CKD-related changes in the human colonic microbial metabolism were largely attributable to dietary restrictions and, to a lesser extent, to loss of renal function. Thus, the plasticity of gut microbiota in response to environmental factors offers a potential avenue for therapeutic interventions aimed at slowing the progression of kidney disease and reducing cardiovascular risk factors in these patients.

#### Dietary Fiber and Prebiotics

Dietary fiber has been defined as edible carbohydrate polymers (with three or more monomeric units), which are not enzymatically digested, hydrolyzed, or absorbed in the small intestine (Jones, 2014). Consumption of a fiber-rich diet has been linked to lower risk of allergy, depression, and cardiovascular disease (Estruch et al., 2013; Rice et al., 2015; Sanchez-Villegas et al., 2015). However, reduction in the intake of dietary fiber, present mostly in fruits and vegetables, is usually recommended to patients with advanced CKD in order to avoid the accumulation of potassium. Unfortunately, as mentioned earlier, fiber deprivation leads to a reduction of intestinal mucus barrier due to the overgrowth of mucin-degrading bacteria (Desai et al., 2016). Furthermore, mucus degradation may enhance pathogen susceptibility and bacteria translocation and exacerbate local and systemic inflammation status (Desai et al., 2016; Andersen et al., 2017). This dilemma can be addressed by a novel approach based on the administration of substances or living organisms aimed at modifying the intestinal bioma. This goal can be achieved by so-called prebiotics, probiotics, symbiotics, or postbiotics.

#### Prebiotics

The use of prebiotics, defined as nondigestible ingredients capable of stimulating the growth and/or activity of health-promoting bacteria, is a widely known strategy to alter the gut microbiota (Bindels et al., 2015). In both healthy and pathological conditions, prebiotics favor the growth of beneficial bacteria (e.g., bifidobacteria and lactobacilli), while suppressing potentially pathogenic microorganisms such as clostridia and enterobacteria (Silk et al., 2009). Inulin supplementation induced a selective increase of “healthy” gut microbiota components, including bifidobacteria, in a randomized double-blind, placebo-controlled, crossover trial (Vandeputte et al., 2017). In a similar study involving obese women, inulin treatment afforded equally beneficial results (Dewulf et al., 2013). Shifts in the gut microbiota composition induced by prebiotics are dependent on their carbon structure (Martinez et al., 2010) and on the microbial enzymatic ability to process them (Koropatkin et al., 2012). Recent data showed that, in patients with type 2 diabetes, dietary fiber intake promoted selective growth of a group of SCFA-producing strains, while most other potential SCFA producers were inhibited or remained unchanged. In addition, the growth of producers of harmful metabolites was diminished. These changes were accompanied by clinical benefits such as lower hemoglobin A_1c_ levels (Zhao et al., 2018).

Besides their potential effect on the generation of SCFAs, plant-based diets exert other effects on metabolites produced by the gut microbiota. For instance, they contain less TMAO precursors compared to an animal-based diet (Richter et al., 2015). Experimental data showed that the consumption of amylose-enriched diet retards the progression of kidney damage through the attenuation of oxidative stress, inflammation, and fibrosis (Vaziri et al., 2014). Additionally, human interventional studies showed that fiber-rich diets and prebiotics ameliorated metabolic disorders (Sola et al., 2007; Aliasgharzadeh et al., 2015), reduced uremic toxins (Meijers et al., 2010b), and delayed the decline in glomerular filtration rate (Pavan, 2016). Moreover, epidemiological studies indicated that high total fiber intake is associated with lower risk of inflammation and mortality in CKD (Krishnamurthy et al., 2012). Meijers et al. (2010b) observed low serum concentrations of IS and p-cresol in hemodialysis patients under dietary prebiotic oligofructose-enriched inulin supplementation. Likewise, the intake of resistant starch improved renal function in CKD (Chiavaroli et al., 2015) and significantly decreased serum concentrations of uremic toxins in hemodialysis patients (Sirich et al., 2014). Extrarenal benefits have also been observed, such as weight loss and improved metabolism of glucose and lipids, lending further support to the therapeutic use of prebiotics (Meijers et al., 2010b; Cani et al., 2007).

#### Probiotics

The Food and Agriculture Organization of the United Nations and the World Health Organization define probiotics as “live microorganisms that when administered in adequate amounts confer a health benefit on the host” (Hill et al., 2014). Probiotics consist of living bacteria (mostly bifidobacteria and lactobacilli species) that can be added to food, drugs, and dietary supplements. As with prebiotics, oral administration of live microorganisms does not significantly change the composition of the indigenous gut microbiota in healthy adults (Kristensen et al., 2016). However, benefits of probiotic therapy have been extensively observed in several pathological scenarios, probably through the generation of bioactive metabolites and immunomodulation (Konstantinov et al., 2008).

Animal studies support the beneficial effects of probiotic therapy in CKD. Administration of *Bacillus pasteurii* and *Lactobacillus sporogenes* reduced blood urea nitrogen (BUN) levels and prolonged the life span of uremic rats (Ranganathan et al., 2005). Treatment with the urease-positive bacterium *Sprosarcina pasteurii* showed similar benefits by removing urea from the gut (Ranganathan et al., 2006). Similar to these findings, treatment of rats with 5/6 renal ablation with *Lactobacillus acidophilus* attenuated inflammation by reducing the serum levels of uremic toxins, such as IS and p-cresyl sulfate, and inflammatory mediators (LPS, C-reactive protein, and IL-6). At the same time, this treatment ameliorated urinary protein excretion and reduced the glomerulosclerosis index (Yoshifuji et al., 2016).

Clinical studies have also demonstrated that probiotic supplementation improves renal function. Simenhoff et al. (Simenhoff et al., 1996) first reported that oral administration of *L. acidophilus* lowered serum levels of the potential uremic toxin dimethylamine and nitrosodimethylamine in hemodialysis patients. Likewise, other studies have observed reduced levels of homocysteine and IS in hemodialysis patients under oral treatment with *Bifidobacterium longum* (Takayama et al., 2003; Taki et al., 2005). A 6-month supplementation with a blend of *L. acidophilus*, *B. longum*, and *Streptococcus thermophilus* ameliorated renal function and the quality of life of 46 patients in stage III or IV of CKD (Ranganathan et al., 2010).

Recently, genetically engineered bacteria have been used to reduce renal inflammation and proinflammatory mediators in intestinal diseases. Bacteria can be genetically modified to sense, kill, or restrain specific pathogens or to produce biomolecules, such as human hormones, ILs, and antibodies within specific organs or tissues (Pinero-Lambea et al., 2015). Oral administration of *Lactococcus lactis* resulted in local delivery of anti-TNF nanobodies at the colon and significantly reduced inflammation in mice with dextran sulfate sodium–induced chronic colitis (Vandenbroucke et al., 2010). Beneficial effects were also reported using a *L. lactis* strain engineered to secrete the anti-inflammatory cytokine IL-10 in Crohn disease (Braat et al., 2006). This cutting-edge therapeutic approach has also been applied in extraintestinal diseases. A strategy using a *L. lactis* strain engineered to secrete proinsulin and IL-10 induced autoantigen-specific long-term tolerance, allowing reversal of established autoimmune diabetes in mice (Takiishi et al., 2012). Engineered bacteria have also been used to emulate urease-producing bacteria. Prakash and Chang (1996) successfully used genetically modified *Escherichia coli* DH5 to remove urea and ammonia, hence lowering BUN in uremic rats.

#### Symbiotics

Combined therapies using prebiotics and probiotics (symbiotics) have been explored in an attempt to alleviate both the toxic effects of uremia and the gut dysbiosis associated with CKD. A randomized double-blind pilot study of CKD stages IV and V reported that a combination of high-molecular-weight inulin, fructo-oligosaccharides, galacto-oligosaccharides, lactobacilli, bifidobacteria, and streptococci altered the stool microbiome, particularly with enrichment of *Bifidobacterium* and depletion of *Ruminococcaceae*, and reduced plasma concentrations of p-cresol (Rossi et al., 2016). Likewise, 4-week treatment with the commercially available symbiotic Probinul-neutro^®^ decreased p-cresol levels in patients with CKD stages III and IV (Guida et al., 2014). Additionally, a therapeutic combination of galacto-oligosaccharides and *Lactobacillus casei* strain Shirota and *Bifidobacterium breve* strain Yakult led to normalization of bowel habits and a decrease of serum p-cresol levels in hemodialysis patients (Nakabayashi et al., 2011).

Despite the benefits observed in a variety of diseases, the success of probiotic-based therapies seems to depend heavily on the pathological condition and on the selected microbial species chosen. For example, treatment with *E. coli* Nissle 1917 is recommended in cases of constipation in adults (Chmielewska and Szajewska, 2010), while *Bacillus coagulans* seems suitable to treat antibiotic-associated diarrhea (Hempel et al., 2012). Further investigation is needed to ensure the safety and the effectiveness of probiotics in different populations and diseases.

#### Postbiotics

The use of postbiotics, also known as metabiotics, has been recently proposed as an adjunctive or alternative therapeutic approach for a healthier gut homeostasis and mucosal immune system. The term *postbiotic* has been coined to designate products from nonviable bacteria or metabolic byproducts from probiotic microorganisms, such as vitamins, SCFAs, cell surface proteins, and enzymes that can affect positively the gut microbiome and the host (Patel and Denning, 2013). According to Shenderov (2013), postbiotics have favorable absorption, metabolism, distribution, and excretion properties, which could indicate a high capacity to elicit favorable biological responses in different organs in the host (Aguilar-Toala et al., 2018). Although the postbiotic therapy has never been applied in CKD, it is noteworthy that many commensal bacteria produce SCFAs, which, as pointed out earlier, exert a number of beneficial effects. Regarding kidney diseases, Andrade-Oliveira et al. (2015) reported that treatment with SCFAs reduces kidney injury induced by ischemia–reperfusion by protecting the renal tissue against inflammation and oxidative stress and by increasing autophagy, reducing apoptosis, and improving mitochondrial biogenesis.

#### Fecal Microbiota Transplantation

Fecal microbiota transplantation (FMT) refers to the introduction of indigenous intestinal microbes from a healthy donor using capsules or colonoscopy into a dysbiotic gut, aiming to restore the microbial community. FMT differs from probiotic therapy in that the donor material is a mixture of undefined microorganisms, including bacteria, yeasts, parasites, and viruses (Hill et al., 2014). Although the first reports date back over 1,700 years (Zhang et al., 2012), FMT is a relatively new therapy for recurrent or refractory *Clostridium difficile* infection (CDI), being moderately recommended as an alternative treatment for a third recurrent CDI after a pulsed vancomycin regimen (Surawicz et al., 2013). The mechanism of action of FMT is unclear. The high rates of resolution of CDI may be due to the infusion of large amounts of bacteria to the gut and/or a variety of metabolites present in the donor sample (Chanyi et al., 2017). FMT has been used in conditions other than CDI, such as metabolic syndrome (Vrieze et al., 2012), autism (Kang et al., 2017), and multiple sclerosis (Makkawi et al., 2018), although the results with irritable bowel syndrome were disappointing (Halkjaer et al., 2018). Clinical studies focusing on the possible beneficial effects of FMT on CKD are lacking. Only anecdotal cases of recurrent infections in a patient with ESRD (Singh et al., 2014) and in a kidney graft recipient (Biehl et al., 2018) have been reported.

Despite their positive effects on the levels of uric acid and BUN, no prebiotic-, probiotic-, or symbiotic-based approaches have been shown to normalize the levels of uremic toxins. Moreover, the long-term impact of these therapies on CKD progression remains unknown due to a number of limitations, such as small sample size and short duration of studies. Larger clinical studies are needed in order to ascertain the possible application of these innovative strategies in the treatment of CKD.

## Repurposed Drugs to Target Renal Inflammation

Recent studies have shown unexpected beneficial effects for repurposed or emerging drugs in the context of AKI and CKD. Several drugs and compounds used to modulate inflammation and immune response in experimental and/or human conditions, such as gout, hypertension, autoimmune diseases, cancer, and organ transplantation, may exert renoprotection by limiting oxidative stress and inflammation. These drugs may exert anti-inflammatory effects by acting on one or more pathogenic pathways such as the RAAS, the innate immune response, and/or the adaptive immunity.

### RAAS Inhibition in Kidney Inflammation

RAAS inhibition is one of the most widely used therapies for the control of hypertension, a leading risk factor for CKD, along with diabetes mellitus. In addition, angiotensin-converting enzyme inhibitors (ACEIs) and angiotensin II receptor blockers (ARBs) are extensively used to prevent or delay the progression of established CKD (Lewis et al., 1993; Brenner et al., 2001; Lewis et al., 2001). Evidence amassed in the past three decades shows that, besides their hemodynamic effects, ACEIs and ARBs directly influence the immune response (Shimada and Yazaki, 1978). Angiotensin II stimulates the expression of inflammatory mediators such as cytokines, chemokines (Ruiz-Ortega et al., 1998; Hisada et al., 1999), growth factors (Kagami et al., 1994; Johnson et al., 1992), and adhesion molecules (Gräfe et al., 1997) in renal parenchymal and immune cells, as well as splenic lymphocyte proliferation (Nataraj et al., 1999). Moreover, intrarenal angiotensin II production may participate in local inflammation (Gilbert et al., 1999; Gonçalves et al., 2004), whereas RAAS blockade was shown to provide hemodynamic-independent renoprotection (Griffin and Bidani, 2006). An extremely high dose of losartan ameliorated renal injury and promoted regression of hypertension and albuminuria in the Nx model by reducing the renal macrophage infiltration and the interstitial angiotensin II expression, rather than by lowering intraglomerular pressure (Fujihara et al., 2005). Losartan also exerted effective renoprotection and anti-inflammatory effects in the streptozotocin diabetes model (Teles et al., 2009). These beneficial effects were markedly amplified when losartan was administered along with hydrochlorothiazide (Fujihara et al., 2007b), even when the combined therapy was started after renal injury was already advanced (Arias et al., 2013). The renoprotective effect of RAAS inhibitors may be partially mediated by limiting activation of the NF-κB pathway (**Figure 2**), known to be activated by angiotensin II in experimental CKD (Ruiz-Ortega et al., 2006).

Few studies have investigated the anti‐inflammatory potential of RAAS-blocking agents in humans. The use of ACEIs is associated with lower plasma TNF-α and C‐reactive protein levels in patients with advanced CKD (Stenvinkel et al., 1999). A comparative study showed that the ACEI ramipril and, especially, the ARB valsartan lowered the IL-6 levels in hemodialysis patients (Gamboa et al., 2012). In addition, losartan treatment prevented the differentiation of monocytes into proinflammatory CD14^+^CD16^+^ cells (Merino et al., 2012) and of oxidative stress–related inflammation (Kayabasi et al., 2013) in hemodialysis patients. However, other studies failed to show blood pressure–independent anti-inflammatory effects of ARBs in hemodialysis patients (Ordaz-Medina et al., 2010; Peters et al., 2017). Thus, further studies are needed to clarify the effect of the RAAS blockers on human renal inflammation.

### Allopurinol Inhibits Activation of the Renal NLRP3 Inflammasome

Hyperuricemia is present in 20% to 35% of patients with CKD (Shan et al., 2010; Lin et al., 2012). Although hyperuricemia is usually asymptomatic, urate crystals may accumulate in tissues such as articular synovium, causing joint inflammation and the symptoms usually associated with gout (Watson, 1899; Ogryzlo et al., 1966; So and Thorens, 2010). Whether hyperuricemia is a real risk factor or just a biomarker of renal and cardiovascular injury is presently unclear. However, recent studies suggest that reducing circulating uric acid slows the progression of CKD (Siu et al., 2006; Goicoechea et al., 2010). Allopurinol, a prodrug of oxipurinol, a xanthine oxidase inhibitor, is widely employed in the treatment of hyperuricemia (Sanders et al., 1997). Besides reducing uric acid production, xanthine oxidase inhibition by allopurinol also diminishes ROS production, thus exerting antioxidant and anti-inflammatory effects (Bakris et al., 1990; Correa-Costa et al., 2011). It is noteworthy that both crystals of uric acid and ROS can activate the NLRP3 inflammasome (Mulay and Anders, 2017). Furthermore, emerging evidence indicates that soluble uric acid is equally capable of activating this inflammasome complex in renal cells and that allopurinol inhibits this process (Correa-Costa et al., 2011; Xiao et al., 2015) (**Figure 2**). Accordingly, NLRP3 inhibition following allopurinol treatment ameliorated renal inflammation and injury in experimental diabetic kidney disease (Kim et al., 2015). In the UUO model, allopurinol reduced the renal content of soluble uric acid and inhibited NLRP3 activation, preventing the progression of proteinuria, as well as renal fibrosis and inflammation (Braga et al., 2017b). In a recent study of the Nx model, we showed that allopurinol reduced soluble renal uric acid and oxidative stress, thus reducing NLRP3 activation and IL-1β levels, as well as tubulointerstitial inflammation and fibrosis (Foresto-Neto et al., 2018).

Inhibition of uric acid synthesis can also exert beneficial effects in human CKD. In a 2-year randomized controlled trial, Goicoechea et al. (Goicoechea et al., 2010) showed that treatment with allopurinol prevented the progressive decline in glomerular filtration rate in patients with CKD of various etiologies. In a *post hoc* analysis of these patients 5 years later, it was shown that long-term treatment with allopurinol not only slowed CKD progression, but also reduced cardiovascular risk (Goicoechea et al., 2015). Similar findings were obtained in a 3-year randomized parallel-controlled study of type 2 diabetic patients with asymptomatic hyperuricemia (Liu et al., 2015). As a whole, these experimental studies and randomized trials reinforce the view that allopurinol can contribute to slow the progression of renal disease.

### NF-κB Inhibition Slows CKD Progression

Inhibition of NF-κB, a major innate immunity pathway, has been proposed as a strategy to reduce inflammation and retard or detain the progression of a number of renal diseases (Sanz et al., 2010). NF-κB activation can be blocked by preventing IkB degradation. The antioxidant compound pyrrolidine dithiocarbamate (PDTC) inhibits IKK, thus preventing NF-ĸB activation (Schreck et al., 1992) (**Figure 2**). In NX model, PDTC treatment inhibited activation of renal NF-ĸB and, ameliorated glomerular and interstitial injury in the Nx model (Fujihara et al., 2007a). In rats receiving adenine overload, PDTC strongly attenuated renal interstitial fibrosis (Okabe et al., 2013). In both studies, PDTC markedly reduced renal infiltration by macrophages, underlining its anti-inflammatory effect. Accordingly, in an experimental model of CKD by type 1 diabetes, renal NF-ĸB was activated in association with increased MCP-1 gene expression and macrophage infiltration as early as 1 month after induction of diabetes mellitus. PDTC reduced renal inflammation at this time point, although its long-term effect on kidney injury was not assessed (Lee et al., 2004).

Interestingly, rats treated with PDTC during lactation developed hypertension as adults, in association with early activation of the RAAS and upregulation of sodium transporters, without renal structural damage or functional impairment (Canale et al., 2011), in contrast with the severe renal damage caused by neonatal losartan (Machado et al., 2008). This finding suggests that the NF-κB system is needed during nephrogenesis for adequate renal control of blood pressure in adult life, but not for nephrogenesis. In a subsequent study (Ávila et al., 2019), rats that had received neonatal PDTC were subjected to uninephrectomy and salt overload in adult life. These animals exhibited marked NF-ĸB activation and developed severe inflammation and injury to glomerular, interstitial, and arterioles. Curiously, treatment of adult rats with losartan inhibited renal NF-κB and attenuated renal injury/inflammation.

Parthenolide is a sesquiterpene lactone that occurs naturally in some plants and has been proposed as an NF-ĸB inhibitor (López-Franco et al., 2006) (**Figure 2**). However, few data are available regarding the effect of parthenolide in kidney diseases. In cisplatin-induced renal damage, parthenolide reduced renal injury and inflammation (Francescato et al., 2007). In the UUO model, parthenolide reduced renal monocyte/macrophage infiltration and attenuated renal injury (Esteban et al., 2004). Although some drugs currently employed in the treatment of kidney diseases possess anti-inflammatory effects and can inhibit renal NF-κB activation (Esteban et al., 2003), no specific NF-κB inhibitor is available for human use. Systemic inhibition of NF-κB may cause substantial adverse effects, since NF-κB is required for adequate nephrogenesis, immune responses, and cell survival. Thus, understanding the mechanisms underlying NF-κB activation and/or inhibition in renal diseases is crucial for the development of more specific regulators, allowing this system to become an important therapeutic target in the quest to prevent the progression of human kidney disease.

## Concluding Remarks

Renal inflammation is central to the development of both AKI and CKD, as well as to the transition of AKI to CKD. Activation of major pathways of innate immunity, such as the NF-ĸB system and the NLRP3 inflammasome, is one of the main factors triggering renal inflammation in both AKI and CKD. The role of the gut microbiota in the pathogenesis of inflammation and its dynamic relationship with renal disease have been unraveled in recent years. New avenues of investigation and new therapeutic perspectives have been opened with the development of suitable NLRP3 and NF-ĸB inhibitors, as well as of strategies centered on the manipulation of the gut microbiota. Additional therapeutic possibilities are provided by the repurposing of old drugs such as allopurinol. New clinical trials will establish the value of these novel approaches for a better comprehension of the mechanisms underlying the pathogenesis of AKI and CKD and for the development of innovative therapeutic strategies in the management of these serious conditions.

## Author Contributions

VA-O, OF-N and IW conceived the figures and wrote the manuscript. RZ and NC conceived the idea, wrote and supervisioned the manuscript.

## Conflict of Interest

The authors declare that the research was conducted in the absence of any commercial or financial relationships that could be construed as a potential conflict of interest.
